# Identification of target groups and individuals for adherence interventions using tree-based prediction models

**DOI:** 10.3389/fphar.2022.1001038

**Published:** 2022-10-19

**Authors:** Johannes Wendl, Andreas Simon, Martin Kistler, Jana Hapfelmeier, Antonius Schneider, Alexander Hapfelmeier

**Affiliations:** ^1^ Institute of General Practice and Health Services Research, School of Medicine, Technical University of Munich, Munich, Germany; ^2^ Vilua Healthcare GmbH, Berlin, Germany; ^3^ Institute of AI and Informatics in Medicine, School of Medicine, Technical University of Munich, Munich, Germany

**Keywords:** adherence, costs, personalized effects, subgroups, model-based trees, model-based random forest

## Abstract

**Background:** In chronically ill patients, medication adherence during implementation can be crucial for treatment success and can decrease health costs. In some populations, regression models do not show this relationship. We aim to estimate subgroup-specific and personalized effects to identify target groups for interventions.

**Methods:** We defined three cohorts of patients with type 1 diabetes (n = 12,713), type 2 diabetes (n = 85,162) and hyperlipidemia (n = 117,485) from German claims data between 2012 and 2015. We estimated the association of adherence during implementation in the first year (proportion of days covered) and mean total costs in the three following years, controlled for sex, age, Charlson’s Comorbidity Index, initial total costs, severity of the disease and surrogates for health behavior. We fitted three different types of models on training data: 1) linear regression models for the overall conditional associations between adherence and costs, 2) model-based trees to identify subgroups of patients with heterogeneous adherence effects, and 3) model-based random forests to estimate personalized adherence effects. To assess the performance of the latter, we conditionally re-estimated the personalized effects using test data, the fixed structure of the forests, and fixed effect estimates of the remaining covariates.

**Results:** 1) our simple linear regression model estimated a positive adherence effect, that is an increase in total costs of 10.73 Euro per PDC-point and year for diabetes type 1, 3.92 Euro for diabetes type 2 and 1.92 Euro for hyperlipidemia (all *p* ≤ 0.001). 2) The model-based tree detected subgroups with negative estimated adherence effects for diabetes type 2 (-1.69 Euro, 24.4% of cohort) and hyperlipidemia (-0.11 Euro, 36.1% and -5.50 Euro, 5.3%). 3) Our model-based random forest estimated personalized adherence effects with a significant proportion (4.2%–24.1%) of negative effects (up to -8.31 Euro). The precision of these estimates was high for diabetes type 2 and hyperlipidemia patients.

**Discussion:** Our approach shows that tree-based models can identify patients with different adherence effects and the precision of personalized effects is measurable. Identified patients can form target groups for adherence-promotion interventions. The method can also be applied to other outcomes such as hospitalization risk to maximize positive health effects of an intervention.

## Introduction

While adherence to medication is believed to play a crucial role in the efficacy of a treatment in many real life settings, its full implementation remains challenging (Dunbar-Jacob and Mortimer-Stephens 2001). This is also the case in chronically ill patients. Hence, a vast variety of different interventions to increase adherence has been suggested (Nieuwlaat et al., 2014). These interventions finally aim to avoid negative health outcomes and/or additional health care costs. Ideally, an intervention can cover its expenditures by avoiding the costs of more severe health developments, which requires higher adherence to be associated with lower total costs and increased health. However, some studies have shown that higher adherence can also be associated with higher total costs, for example when additional drug costs exceed savings in inpatient and outpatient costs (Iuga and McGuire 2014; Cutler et al., 2018).

These and many other studies model the relationship of adherence and costs in a study population and estimate an overall effect of adherence. For example, the usually applied linear regression model estimates the average effect of adherence for the population. In our case, in contrast, we assumed that there might be individual effects that express in different size or even sign. For example, even when the overall effect is positive, there might still be some patients with a negative effect of adherence on costs. We therefore exploited methods provided by the increasing field of personalized medicine research (Weisberg 2015). The objective was to model treatment effects depending on patients’ characteristics, to explore the stratified and personalized effects of adherence.

The identification of patients with a negative relation between adherence and costs can be an aspect of selecting a target group for an intervention. This has been considered to be important for the efficiency of an intervention and can help to reduce the number of people who need to be targeted (Fuchs 2008). An intervention often is applied to a specific group where the need or the expected effect is highest. One area of application is to identify a subgroup of patients of which we can expect the avoided costs (by increased adherence) to be greater than the additional costs of its expenditures.

The identification of these subgroups can be defined theoretically in a hypothesis-driven approach. So far, to our knowledge there are only two studies about subgroup-specific effects in adherence-costs relationship. One of it, by Roebuck et al. (2015) analyzed a population of Medicaid enrollees with low income. They segmented the population according to their basis of eligibility for Medicaid in blind or disabled, other adults, and children and modeled these subpopulations separately. In a preceding study, Roebuck et al. (2011) used interaction effects to estimate age and sex-specific effects of adherence within a single model. The main disadvantage of this approach is that it either requires prior knowledge or strongly depends on assumptions about the functional form of the underlying effect. A major advantage is that subgroups can be compared directly when modeled simultaneously.

The other main approach for the identification of subgroups is data-driven, often by using modern statistical methods to automatically detect subgroups in the data structure. For this purpose, we use decision-tree-based methods to detect subgroups and to estimate subgroup-specific regression models of adherence effects (Seibold et al., 2016). Respective model-based random forests can even be exploited to differentiate between effects on the individual patient level (Seibold et al., 2018). The goal of the present paper is threefold: 1) evaluate the overall relation between adherence and costs, 2) identify subgroups with significantly better response to medication adherence, and 3) provide a model to estimate a patient’s individual conditional adherence effects. To reach our research goals, we focused on the development and the application of novel predictive models which transfer the regression approach to a machine learning procedure. We specifically do not want to propose or apply a specific intervention to a group of patients. Instead, we suggest an approach to identify target groups and individuals to maximize the effect of an intervention, given that this intervention is able to increase adherence.

## Materials and methods

### Data

We used a database of German claims data of the years 2007–2016. It contains over 3.5 million statutory insured persons with data about their age, sex, charges, diagnoses coded according to the German modification of the international classification of diseases (ICD-10-GM), filled prescription drugs by date, package size, Anatomical Therapeutic Chemical (ATC) classification code and Defined Daily Dose (DDD) according to WHO Collaborating Centre for Drug Statistics Methodology (2021). Also, information about the participation in one of six disease management programs (DMP) for asthma, breast cancer, chronic obstructive pulmonary disease, type 1 diabetes, type 2 diabetes and coronary heart disease are available for all persons in the complete period.

### Study population

We extracted data of the latest 4.5 fully available years (July 2011 until December 2015) and defined data of 2012 as baseline and the years 2013–2015 as follow-up. Only patients with year-round coverage in these years were considered in the present study. We focused on patients with chronic diseases to observe the adherence-costs relationship over a longer period of time. Patients with at least one diagnosis within each observational year of type 1 diabetes (T1D: ICD-10-GM code E10), type 2 diabetes (T2D: E11), or hyperlipidemia (E78) were selected for three cohorts. Patients with multiple of the diagnoses of interest were selected for multiple cohorts.

We excluded patient years with excess costs (top 5% total charges of each cohort) to avoid costs which are rather influenced by expensive treatments like dialysis or severe accidents than by the chronic disease itself. These patient years might distort the estimation of adherence effects. Moreover, all patients having no data or fills of corresponding prescription drugs in 2012 were excluded. See Supplementary Table S1 for the definition of diseases and drugs.

### Definition of variables

The outcome variable was mean annual total costs in follow up years. We used a time lag between adherence measured at baseline and costs measured during follow-up to avoid reverse causality. Reverse causality might appear when major adverse health events and hospitalization increase costs and likewise result in initiation of drug therapy and influence adherence (Stuart et al., 2014; Roebuck et al., 2015). In an earlier, not yet published work, we found that the mean annual total costs are appropriate for our approach. Therefore, we summed up all patient’s individual charges per year and calculated the mean of the follow-up years 2013–2015 with all prices converted to Euros 2015 according to the annual inflation of the healthcare sector as stated by the German Federal Statistical Office (Statistisches Bundesamt (Destatis) 2022).

In this paper, we focus on medication adherence during treatment as the “extent to which a patient’s actual dosing corresponds to the prescribed dosing regimen” (Vrijens et al., 2012). Adherence at baseline year 2012 was defined as proportion of days covered (PDC) by any diagnosis specific medication. We used the PDC, because even in case of oversupply—in contrast to the often used medication possession ratio (MPR)—it is still limited to the range 0–100. To calculate the PDC, we counted a day as covered when at least one dose of any diagnosis specific drug, distinguished by its ATC code (WHO Collaborating Centre for Drug Statistics Methodology 2021), was available to the patient. We assumed this was the case 1) within the period after the prescription fill for the number of days calculated by total package size divided by the DDD (WHO Collaborating Centre for Drug Statistics Methodology 2021) or 2) during hospitalization if the patient had filled the same drug within 3 months before or after the hospital visit. In both cases we proportionally considered fills and hospitalizations in the last half of 2011 if the covered days reached into 2012. We divided the number of covered days by the number of days between the first covered day and the last day of 2012 and used the continuous PDC—instead of a dichotomized PDC—to avoid loss of information and the risk of bias (Tueller et al., 2016).

We further extracted some baseline characteristics, such as age and sex as sociodemographic variables, Charlson’s Comorbidity Index (CCI) in its ICD-10 version with updated weights (Charlson et al., 1987; Quan et al., 2005, 2011) and initial total costs to reflect the general health status, and a two- or three-level severity variable of the chronic diseases based on treatment guidelines and prescription drug fills to include the degree of severity of a given disease (Supplementary Table S2). Furthermore, participation in any DMP and influenza vaccination at baseline were used as proxys for health behavior which has been discussed to be an important confounder but is not directly available in the analyzed claims data (Shrank et al., 2011).

### Statistical analysis

All statistical analyses were performed in R version 3.6.2 (R Core Team 2019). Hypothesis testing was performed at exploratory two-sided 5% levels of significance. We split our cohorts into a training and a test data set of 50% each and fitted the different types of models on the training data set. The test data set was used to evaluate the model-based random forest.

#### Linear regression

To estimate the overall conditional effect of adherence on total costs we used a multivariable linear regression model with mean annual total costs as outcome; adherence as main predictor; and age, sex, CCI, initial total costs, severity, participation in any DMP, and influenza vaccination as covariates. The linear regression model assumes the estimated adherence effect is constant for all patients.

#### Model based decision tree

To identify potential subgroups of patients with different estimated conditional adherence effects, we used a model-based tree in the framework of model-based recursive partitioning (Seibold et al., 2016). This method builds a decision tree which splits the cohort into subgroups by pre-specified candidate partitioning variables. A split is performed when the model parameters are found to be statistically significant dependent on any of the partitioning variables. Then, an optimal cut-point of the partitioning variable is determined as it maximizes the sum of the likelihoods of the two resulting models fit to the respective subsets of the data. This procedure of refitting models to subsets of the data continues recursively until no further statistically significant associations are found or no further splits are possible because of restrictions on the minimally required subgroup sizes.

In our case, we used a linear regression model as the base model and searched for subgroups that differ in the estimated effect of adherence on total costs. In the model-based tree, we specified initial costs, age, CCI and severity as candidate partitioning variables because we expected them to potentially modify the effect of adherence. The procedure thereby implicitly models interactions between the partitioning variables and adherence. We further defined the minimal subset size (terminal node size) to 5% of the cohort to avoid subgroups that are too small for interventions in practice.

Model-based trees again assume the estimated effect is constant for patients within each subgroup, while this must not be true for all patients as a whole (Seibold et al., 2018). The effect is essentially modeled as a step function of the selected partitioning variables. This assumption may be too restrictive when the interaction function is smooth and personalized effect estimates are more appropriate.

#### Model based random forest

To estimate personalized effects, we used weighted linear regression models derived from a model-based random forest (Seibold et al., 2018). The random forest is an ensemble of the aforementioned model-based trees fitted to random samples of the data and random selections of the partitioning variables. The procedure provides a natural measure of similarity between observations. Therefore, one can count the number of times each pair of observations is allocated to the same subset in each of the many trees of the forest. For example, in a forest consisting of 500 trees, patient A could be in the same defined subgroup as patient B or patient C in 250 and 300 trees. Fitting a personalized model for patient A would consequently assign weights of 1, 250/500 = 0.5 and 300/500 = 0.6 to the observations of patient A, B and C in the data. The linear regression models are otherwise specified as outlined above. We fitted the model-based random forest by the implementation of transformation forests introduced by Hothorn and Zeileis (2021). We applied different specifications of the minimal subset size (terminal node size) of the trees (n_s_ = 200, 500 or 1,000) to allow three levels of similarity, with larger subgroups consisting of less similar patients and *vice versa*.

For further investigation of estimated personalized effects, we plotted partial dependence plots which show the relation of the partitioning variables age, initial costs, CCI, and severity to the personalized adherence effects by means of a smooth curve (Seibold et al., 2018). We also developed a new calibration-like approach. Therefore, we conditionally re-estimated the personalized adherence effects by using the test data, the fixed structure of the forests and fixed effect estimates of the remaining covariates of the model. We fixed the estimates of the covariates as we subtracted their estimated effects from the outcome before re-estimating the adherence effect in the test data. For a subsample of 1,000 patients, we compared the effects estimated by the forest to the conditional effects re-estimated on test data. We used univariate regression models of these two estimates to explore model calibration and to assess the precision the estimates. Because the scatter plot of the two estimates showed deviations from a linear fit, we fitted three GAMLSS regression models with different assumptions (Stasinopoulos et al., 2017). The first assumes a linear fit, the second assumes a nonlinear fit estimated with cubic splines, and the third additionally models the variance with cubic splines.

The 95% prediction intervals of the regression models were used to identify patients of which we can expect a negative adherence effect with the given certainty based on the respective personalized effect estimation of the forest. When the upper limit of the prediction interval is negative, we can expect a negative personalized adherence effect on costs with the corresponding certainty. We henceforth call them certainty-controlled personalized estimated effects.

## Results

**FIGURE 1:**
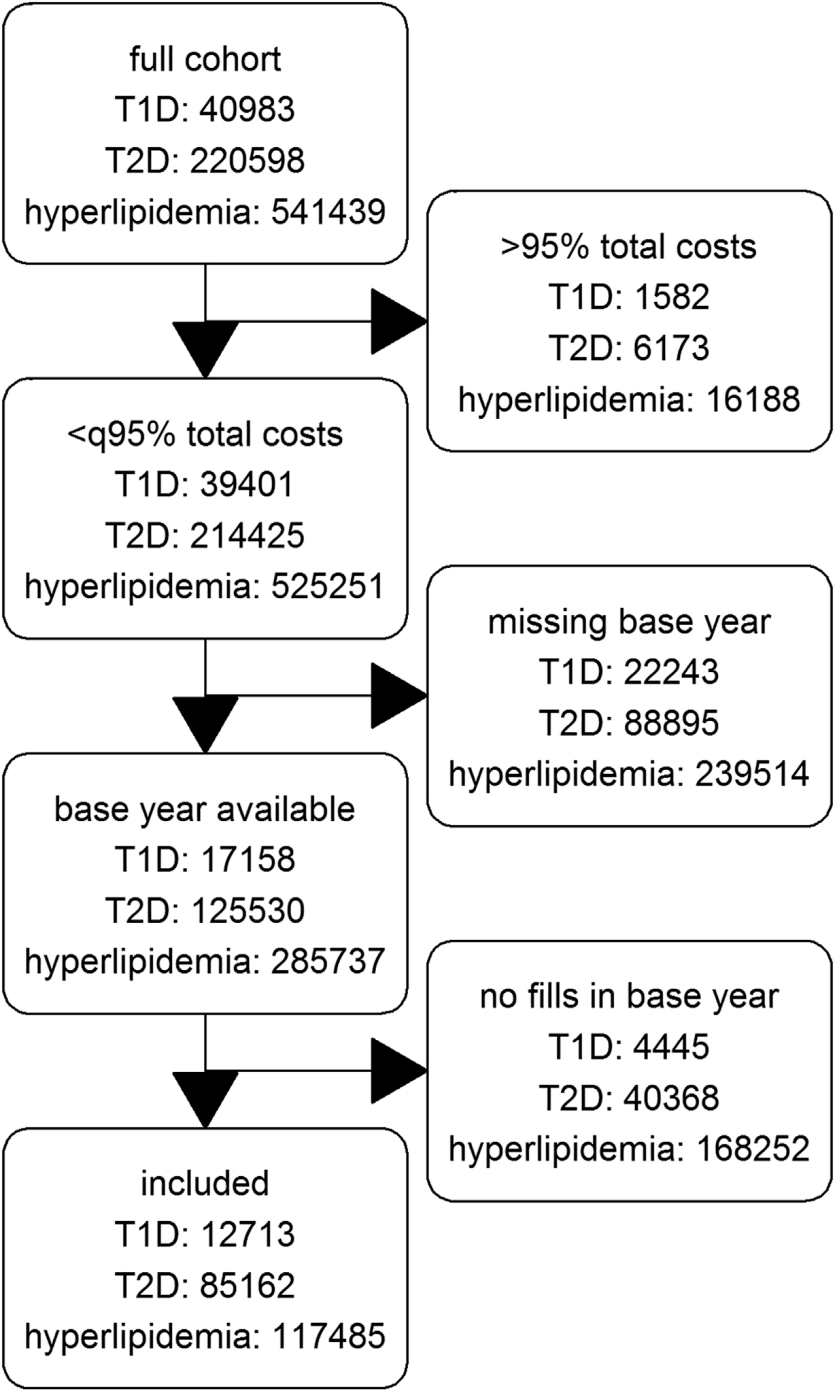
Flowchart of cohorts.

Of the 2,644,212 patients with at least one year-round coverage between 2012 and 2015 in the database, we finally include 12,713 patients with T1D, 85,162 patients with T2D and 117,485 patients with hyperlipidemia. Figure 1 shows a flow chart of included, and excluded patients per diagnose.

In T1D and T2D patients, the median PDC was higher than in hyperlipidemia patients with 88 and 84 compared to 64 respectively. Being extremely left skewed, 62% and 54% of the diabetes patients had a PDC higher than 80. In the hyperlipiedemia cohort, only 30% had a PDC higher than 80 and the distribution was more balanced. The total costs of all cohorts were right skewed. The median of the mean annual total costs were highest in T1D with 4,848 Euro followed by T2D with 3,404 Euro and hyperlipidemia with 2,329 Euro. These and further descriptive statistics are given in Table 1.

**TABLE 1:** Descriptive summary statistics of cohorts of 3 chronic diseases: Median (IQR) for continuous and absolute (relative) frequencies for categorical variables.

Variable	T1D^a^	T2D^b^	hyperlipidemia
PDC^c^	88.3 (68.0, 99.7)	83.6 (53.8, 98.1)	63.9 (37.6, 88.2)
Female (Yes)	6,155 (48.4%)	44,709 (52.5%)	61,159 (52.1%)
Age	59.0 (44.0, 72.0)	68.0 (58.0, 76.0)	69.0 (59.0, 76.0)
Severity			
light	4,892 (38.5%)	28,824 (33.8%)	107,061 (91.1%)
medium	7,821 (61.5%)	26,885 (31.6%)	10,424 (8.9%)
severe	-	29,453 (34.6%)	-
Initial Costs	4,183.4 (2,662.0, 7,325.8)	2,534.5 (1,247.1, 5,305.1)	1,792.8 (847.7, 4,147.9)
CCI^d^	3.0 (2.0, 5.0)	3.0 (2.0, 5.0)	2.0 (1.0, 4.0)
DMP (Yes)^e^	9,537 (75.0%)	63,537 (74.6%)	47,016 (40.0%)
Vaccination (Yes)	3,222 (25.3%)	26,228 (30.8%)	35,733 (30.4%)
Total Costs	4,847.9 (3,089.6, 8,159.1)	3,403.7 (1,730.0, 6,489.4)	2,328.7 (1,126.3, 4,755.6)

^a^ Type 1 Diabetes.

^b^ Type 2 Diabetes.

^c^ Proportion of Days Covered.

^d^ Charlson’s Comorbidity Index.

^e^ Disease Management Program.

The simple linear regression model estimated a positive adherence effect on total costs of 10.73 Euro per PDC-point and year for T1D, 3.92 Euro for T2D and 1.92 Euro for hyperlipidemia (all *p* ≤ 0.001) when we controlled for age, sex, CCI, initial total costs, severity, participation in any DMP and influenza vaccination (Supplementary Table S3). In all three cohorts, higher adherence was associated with higher total costs.

When we applied model-based trees, we detected subgroups defined by initial total costs, CCI and age in all three cohorts (Table 2). Of the candidate partitioning variables, only severity was never used to define the subgroups. T1D patients were split in three subgroups by initial total costs: in the largest subgroup (77.5% of T1D patients) with initial total costs lower than 7,813 Euro, the subgroup-specific estimated effect of adherence on total costs was lowest with 4.21 Euro per PDC-point and year. It was therefore lower than the overall effect, but still positive. The other two subgroups defined by higher initial costs had an adherence effect above average. Due to the small sample size of the T1D cohort, the effect in all subgroups did not reach statistical significance.

**TABLE 2:** Adherence effect estimates and subgroups detected by model-based decision trees.

Diagnosis	Subgroup	Estimate	*p*-value	n (%)
T1D	initial costs <= 15,996 and initial costs <= 7,813	4.21	0.069	4,927 (77.5)
T1D	initial costs <= 15,996 and initial costs >7,813	13.41	0.062	1,055 (16.6)
T1D	initial costs >15,996	16.45	0.248	374 (5.9)
T2D	initial costs <= 7,307 and initial costs <= 3,130 and age <= 63	-1.69	0.087	10,394 (24.4)
T2D	initial costs <= 7,307 and initial costs <= 3,130 and age >63	1.86	0.069	14,154 (33.2)
T2D	initial costs <= 7,307 and initial costs >3,130 and age <= 76	9.17	0.000	7,930 (18.6)
T2D	initial costs <= 7,307 and initial costs >3,130 and age >76	1.60	0.612	2,851 (6.7)
T2D	initial costs >7,307	6.21	0.012	7,252 (17.0)
hyperlipidemia	initial costs <= 3,179 and initial costs <= 1,563 and cci <= 2	-0.11	0.819	21,233 (36.1)
hyperlipidemia	initial costs <= 3,179 and initial costs <= 1,563 and cci >2	2.98	0.009	5,661 (9.6)
hyperlipidemia	initial costs <= 3,179 and initial costs >1,563 and age <= 60	-5.50	0.000	3,125 (5.3)
hyperlipidemia	initial costs <= 3,179 and initial costs >1,563 and age >60	3.60	0.000	9,593 (16.3)
hyperlipidemia	initial costs >3,179	2.03	0.027	19,130 (32.6)

T2D patients were split in five subgroups by initial total costs and age. Patients with lower initial total costs than 3,130 Euro and an age of 63 or younger formed a large subgroup (24.4%) in which higher adherence was associated with lower total costs with an estimated effect of -1.69 Euro per PDC-point and year. Of the other subgroups, two had an adherence effect below average. The effects in all of these mentioned subgroups were not statistically significant.

In hyperlipidemia patients we detected five subgroups defined by initial costs, age and CCI. In two subgroups higher adherence was associated with lower costs. In a large subgroup (36.1%) of patients with initial costs lower than 1,563 Euro and a CCI of two or lower, the estimated effect was -0.11 Euro per PDC-point and year. In another small subgroup (5.3%) with medium initial costs between 1,563 Euro and 3,179 Euro and an age of 60 or younger, the estimated effect was -5.50 Euro. In the latter subgroups the effect was statistically significant. The other subgroups had an adherence effect higher than the overall effect. Table 2 gives an overview of all subgroups. A graphical representation of the trees can be found in the Supplementary Figures S1–S3.

The model-based random forest estimated a significant proportion of negative personalized adherence effects (Table 3). These proportions ranged from 0.0% to 4.2% for T1D, 6.0%–20.5% for T2D and 16.6%–24.1% for hyperlipidemia, depending on the level of similarity, which is controlled by the minimally required subset size (n_s_) in the forest models. We estimated personalized adherence effects of up to -1.17 Euro, -7.45 Euro, and -8.31 Euro, respectively. For higher levels of similarity—and therefore lower subset sizes—we obtained more diverse personalized effect estimates and, in consequence, a larger proportion of negative effects.

**TABLE 3:** Proportion (range) of negative personalized estimated effects of adherence on costs.

Diagnosis	Model	Estimated effect	Certainty-controlled estimated effect
T1D	n_s_ = 200	4.2% (-1.17; -0.06)	6.3% (-1.17; 0.22)
	n_s_ = 500	0.0% (-)	0.0% (-)
	n_s_ = 1,000	0.0% (-)	0.0% (-)
T2D	n_s_ = 200	20.5% (-7.45; -0.01)	3.9% (-7.45; -3.21)
	n_s_ = 500	10.9% (-3.53; -0.02)	3.9% (-3.53; -1.18)
	n_s_ = 1,000	6.0% (-0.77; 0.00)	0.6% (-0.77; -0.59)
hyperlipidemia	n_s_ = 200	24.1% (-8.31; -0.01)	8.3% (-8.31; -1.72)
	n_s_ = 500	21.3% (-3.50; -0.01)	5.0% (-3.50; -1.50)
	n_s_ = 1,000	16.6% (-1.43; 0.00)	4.0% (-1.43; -0.96)

**FIGURE 2:**
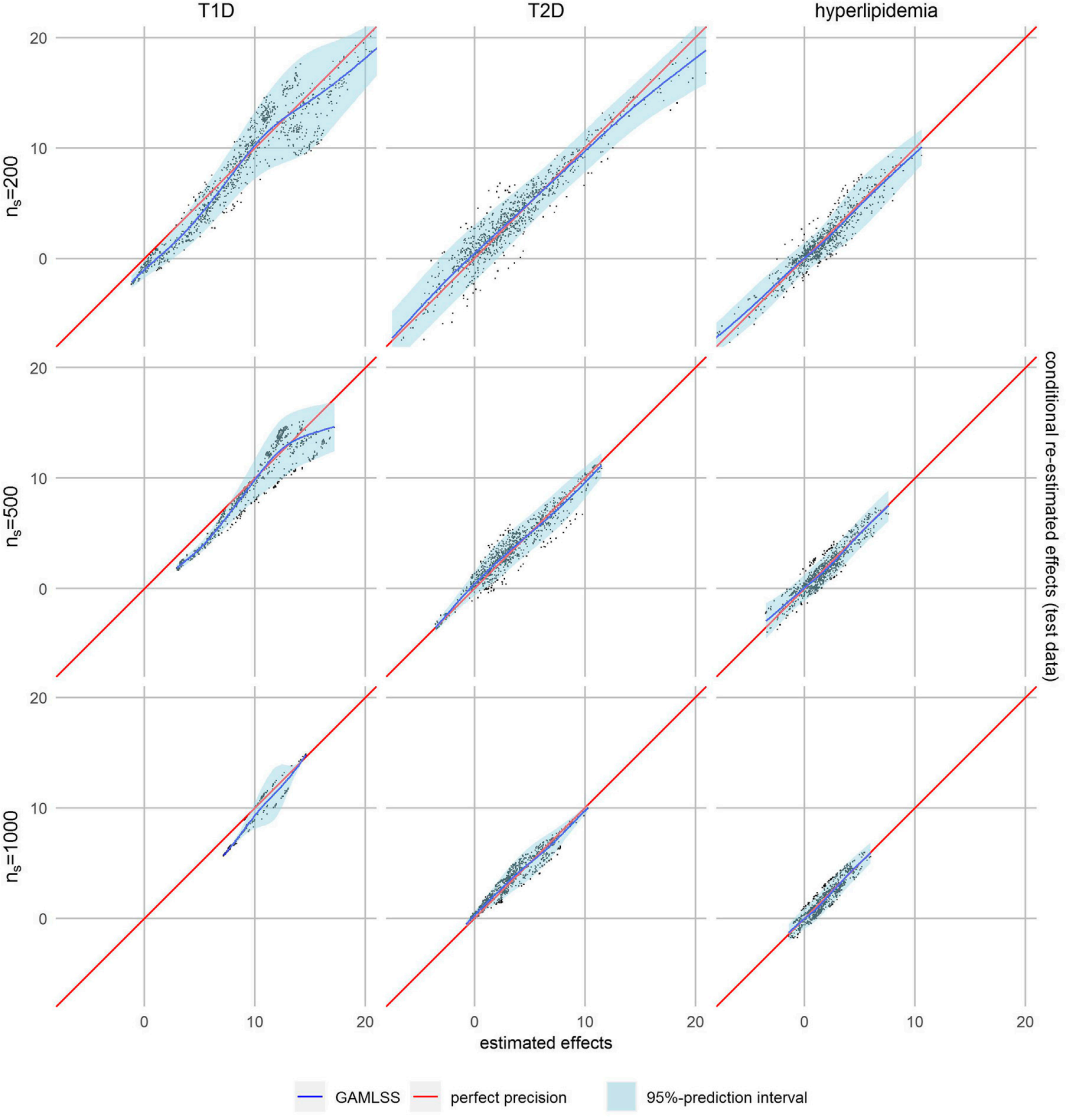
Calibration plots with non-linear fit (blue line) and 95% prediction interval (light blue area).

However, smaller subset sizes may also lead to increased variability and therefore decreased precision in effect estimation. We therefore applied our calibration-like approach to assess the quality of effect estimation. The estimated personalized effects are plotted against the conditional ones re-fitted on test data, while regression models were used to assess their relation. A visual comparison of model fits showed the best fit for the GAMLSS model with a nonlinear fit of mean and variance in almost all cases (Supplementary Figures S4, S5). The calibration plot in Figure 2, where perfect precision is illustrated by a diagonal red line, shows that the effect estimates for T2D and hyperlipidemia patients were well-calibrated, which is not the case for those for T1D patients. In the latter, lower estimated effects seem to be overestimated because the regression curve of the GAMLSS model (blue line) is systematically lower than expected in case of perfect precision (red line). The regression curve of T2D and hyperlipidemia is closer to perfect precision.

The 95%-prediction interval of the GAMLSS regression models (light blue area) identifies 0.0%–6.3% of patients with T1D, 0.6%–3.9% of patients with T2D and 4.0%–8.3% of patients with hyperlipidemia with a negative certainty-controlled personalized estimated effect. For high level of similarity we can expect a negative adherence effect with the given certainty when the estimated effect of the forest was lower than 0.22 Euro, -3.21 Euro and -1.72 Euro, respectively. The value for T1D is counterintuitivly positive because this model is not well calibrated. Again, the variance of re-estimated effects is higher and the prediction intervals wider—indicating lower precision—if the defined level of similarity was higher.

**FIGURE 3:**
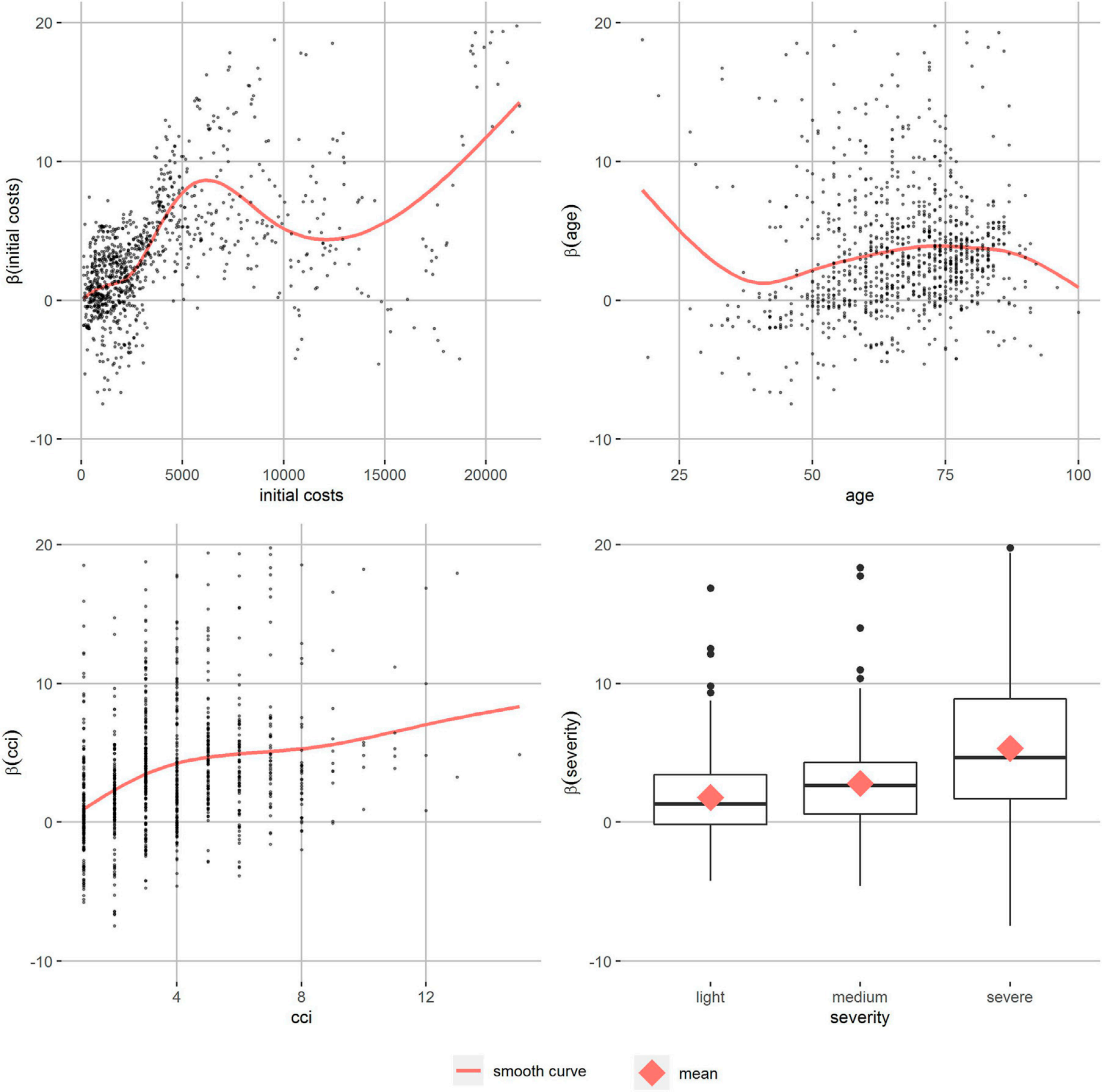
Partial dependence plots of T2D patients with high level of similarity.

The partial dependence plots of T2D with a high level of similarity (Figure 3) show the relation of the personalized effect estimates to the partitioning variables. They increase continuously by initial costs until around 6,000 Euro. The data gets more sparse and the smooth curve starts fluctuating. The age effect on the personalized effect estimates also increases in the main age groups between 50 an 80, as well as the CCI’s effect. More severe T2D patients’ effect estimates are higher on average. Patients with T1D and hyperlipidemia show similar patterns (Supplementary Figures S6–S13). In T1D patients, the increase of personalized effects by initial costs can be observed at higher initial costs and there are no differences in severity. For hyperlipidemia patients, there was almost no effect of initial costs and a reverse severity effect. In all partial dependence plots, apart from some age groups of hyperlipidemia patients, the smoothed curve of the adherence effects is positive. Comparison of different levels of similarity showed similar patterns, but the between-person differences were smaller as expected.

## Discussion

In T1D, T2D and hyperlipidemia patients, model-based trees and forests often identified patients with negative estimated effects of adherence on costs, while simple multivariable linear regression models showed a positive association overall. In general, patients with negative estimated effects of adherence on costs were healthier and younger. Our approach shows that tree-based models can identify patients with different effects up to the individual level, while the quality of effect estimation of such models can be assessed simultaneously.

Using model-based trees, we stratified the overall effects estimated by the linear regression models and detected large subgroups with an estimated effect below average in all cohorts. In T2D and hyperlipidemia, there were subgroups with a negative estimated effect of adherence on costs, which consists of around 25% and 40% of our cohort respectively. This was not the case in T1D in which the subgroup with the lowest adherence effect still had a positive association of adherence and costs. With few exceptions, the effect of adherence on costs is lower in younger patients, as well as when initial costs are low and the CCI indicates less comorbidities. Although the cut-points are model-specific, it seems like healthier patients have a lower, and in some cases even negative, effect of adherence on costs.

Going beyond stratified effects towards personalized effects, the model-based random forest also identified patients with an estimated adherence effect below average. Here, in all three cohorts up to around 5%, 20% and 25% of patients could be identified as having a negative estimated effect of adherence on costs. Further investigation of the personalized effects showed a similar pattern as observed in the model-based decision trees. The effects of adherence on costs increase with higher initial costs, more comorbidities and higher age. Again, differences by severity of disease were inconsistent and comparatively low. In addition, it seems like there is no single variable which explains negative individual differences alone, but it might be a combination of different characteristics—like low initial costs and few comorbidities—that make a negative effect of adherence on costs more likely.

Despite the similarities between the three diagnoses, we also found some differences. In the T1D cohort, no or considerably fewer patients were identified having a negative adherence effect. The reason might be that the overall effect was comparatively high, but also—as smallest cohort—sample size may have restricted detection of differences in the data structure. The cohort of hyperlipidemia patients had less initial costs, lower CCI, and—according to our classification of severity—mainly milder forms of the disease. Here, initial costs do not substantially explain differences of personalized effects in the random forest. Furthermore, the subgroup with the lowest estimated effect consists of medium initial costs.

In the only study with an interaction model, Roebuck et al. (2011) found statistically significant age differences on the effect of adherence in dyslipidemia and diabetes patients with higher cost savings in patients older than 65 and no statistically significant sex differences on the effect in these populations. This is in contrast to our findings where younger patients had costs savings. Hence, the applied methods to stratify and personalize the effect estimates of adherence on costs are only the first step and further studies are necessary to explain the effect of the identified patients’ characteristics and differences between diagnoses.

With our calibration-like approach, we were able to assess the quality of the effect estimation by model-based random forests. In such models, the estimated personalized effects depend not only on the structure of the fitted forest, but also on the data used to fit the personalized models. We exchanged this data by using test data to assess the quality of effect estimation. Visual comparison of the effect estimates obtained from training data and test data—conditional on the forest structure and effect estimates of other covariates—show whether the effect estimates are precise. Precision was reduced in the models for T1D patients, where we observed deviations between the two estimated effects. Hence, the results should be interpreted with caution. In the other two cohorts, the personalized effect estimates were more precise. Moreover, the prediction interval of the regression models of the two estimates show the range of the expected personalized effects if fitted on test data with a certainty of 95%. We identified patients with negative estimated effect also when using these certainty-controlled estimated effects.

Of course, there are some limitations to the present study. The training as well as the test data came from the same population and the generalization of the results is limited. An external validation would solve this problem and can make use of the proposed method of calibration. In Germany, health insurance is compulsory and the stationary insurances cover almost 90% of the total population (Statistisches Bundesamt (Destatis) 2020). Therefore, we expect our data to be generalizable for Germany and with some limitations also for other countries. Nevertheless, we would recommend training the models on data as similar as possible to the final target population.

In the model-based random forests, we observed a trade-off between the variance and precision of estimates depending on the defined level of similarity between patients. With decreasing minimal subset size of the trees, in other words increasing level of similarity, the variance of personalized effect estimates increases for training and test data. This results in a larger proportion of negative effect estimates on the one hand. On the other hand, the prediction intervals are wider and thus there is a smaller proportion of certainty-controlled negative effect estimate. Further research on this aspect with the aim to identify an optimal value is necessary.

Other methods to investigate the effect of patients’ characteristics on the effect of the main predictor are available. A linear interaction of a continuous covariate and the main predictor gives a robust estimation of the effect in many scenarios, especially when the true underlying effect is linear, and outperforms common approaches like categorization by the median (Haller et al., 2019). An advantage of the applied methods compared to a regression model with interaction, is that they do not only automatically select partitioning variables, but also select their optimal cut-points to define the subgroups (Seibold et al., 2016). In their study, Roebuck et al. (2011) chose a cut-point of 65 for age without a reported justification and it is unclear how a different cut-point would have influenced his results. Especially when visualized graphically, model-based trees are easy to interpret (Zeileis et al., 2008). The structure of the tree and the underlying decision rules are both less complex than higher order interactions of a regression model and more flexible than other available methods (Seibold et al., 2016). An important disadvantage of decision trees is their instability, even when the data only changes slightly (James et al., 2021). However, this is expected to be less of a problem given the large sample size in the present study. In this respect, random forests are more stable compared to a single tree due to the large amount of included trees. But because the effects are calculated from the ensemble of all trees, the model cannot be interpreted directly anymore (Hastie et al., 2009). Instead, partial dependence plots can give insights into some properties of the forest and its effects. In our case, the main advantage of model-based forests is their ability to estimate personalized effects (Seibold et al., 2018).

The identified patients can be assigned to target groups for adherence-promotion interventions with the aim to increase health and decrease associated costs. The proposed method can also be applied to predict other outcomes such as hospitalization risk to maximize positive health effects of an intervention. Originally developed for clinical trails, the methods can also be applied to directly detect subgroups and personalized effects during an intervention study.

## Data Availability

The data analyzed in this study is subject to the following licenses/restrictions: Requests to access these datasets should be directed to johannes.wendl@mri.tum.de.
