# Supplementary material for: Identification of target groups and individuals for adherence interventions using tree-based prediction models
Source: Front Pharmacol. 2022 Oct 19;13:1001038. doi: 10.3389/fphar.2022.1001038 (PMC9627286; doi:10.3389/fphar.2022.1001038)
Supplement: Supplementary file 1 [file DataSheet1.PDF]

Identification of target groups and individuals for adherence interventions using tree-based prediction models

Frontiers in Pharmacology

Johannes Wendl*[1], Andreas Simon[2], Martin Kistler[2],
Jana Hapfelmeier[2], Antonius Schneider[1], Alexander Hapfelmeier[1,3]

Sep 15, 2022

[1]Institute of General Practice and Health Services Research, School of Medicine, Technical University of Munich, Germany
[2]Vilua Healthcare GmbH, Berlin, Germany
[3]Institute of AI and Informatics in Medicine, School of Medicine, Technical University of Munich, Germany

*Corresponding Author: Johannes Wendl (johannes.wendl@mri.tum.de)

Supplementary Table 1

Table 1: Definitions of Diagnosis and Corresponding Drugs.

Diagnosis	ICD-10 Code	Corresponding ATC Codes
Type 1 Diabetes	E10*	A10A*
Type 2 Diabetes	E11*	A10*
Hyperlipidemia	E78*	C10AA*, C10AB*, C10AC*, C10AD*, C10BA*, C10BX*

* and sublevels

Supplementary Table 2

Table 2: Definitions of Severity by Diagnosis.

Diagnosis	Light	Medium	Severe	Reference
Type 1 Diabetes	A10AB* (Insulins and analogues for injection, fast-acting) OR A10AC* (Insulins and analogues for injection, intermediate-acting) OR A10AF* (Insulins and analogues for inhalation)	A10AD* (Insulins and analogues for injection, intermediate- or long-acting combined with fast-acting) OR A10AE* (Insulins and analogues for injection, long-acting)	not defined	(DDG 2018)
Type 2 Diabetes	only metformin (A10BA02)	other drugs used in diabetes (A10*) without insulins and analogues (A10A*)	insulins and analogues (A10A*)	(Bundes-ärzte-kammer, KBV, and AWMF 2021)
Hyperlipidemia	C10AA* (HMG CoA reductase inhibitors)	C10AB* (Fibrates) OR C10AC* (Bile acid sequestrants) OR C10BA* (Combinations of various lipid modifying agents) OR C10BX* (Lipid modifying agents in combination with other drugs)	not defined	(Mach et al. 2020)

Note:

Only ATC Codes listed in Supplementary Table 1 are considered

* and sublevels

Supplementary Table 3

Table 3: Effect Estimates (Standard Errors) of Linear Regression Models of Total Costs.

	T1D	T2D	Hyperlipidemia
Intercept	841.43**	−669.08***	−555.77***
	(267.56)	(102.87)	(70.81)
PDC	10.73***	3.92***	1.92***
	(2.29)	(0.70)	(0.40)
Severity (Medium)	−32.51	333.35***	241.29***
	(102.63)	(43.15)	(38.88)
Severity (Severe)		1,340.22***	
		(45.77)	
Initial Costs	0.48***	0.40***	0.35***
	(0.01)	(0.004)	(0.003)
Female	347.38***	126.24***	114.78***
	(100.94)	(33.18)	(22.75)
Age	16.30***	32.11***	28.91***
	(3.15)	(1.41)	(1.04)
CCI	225.38***	206.23***	243.98***
	(25.79)	(8.16)	(5.85)
DMP	−21.77	−144.10***	304.89***
	(117.03)	(37.81)	(24.46)
Vaccination	160.52	106.13**	65.23**
	(118.67)	(36.04)	(24.56)
Observations	6,356	42,581	58,742
Adjusted R^2	0.32	0.33	0.28
Note:			*p<0.05; **p<0.01; ***p<0.001

Supplementary Figure 1

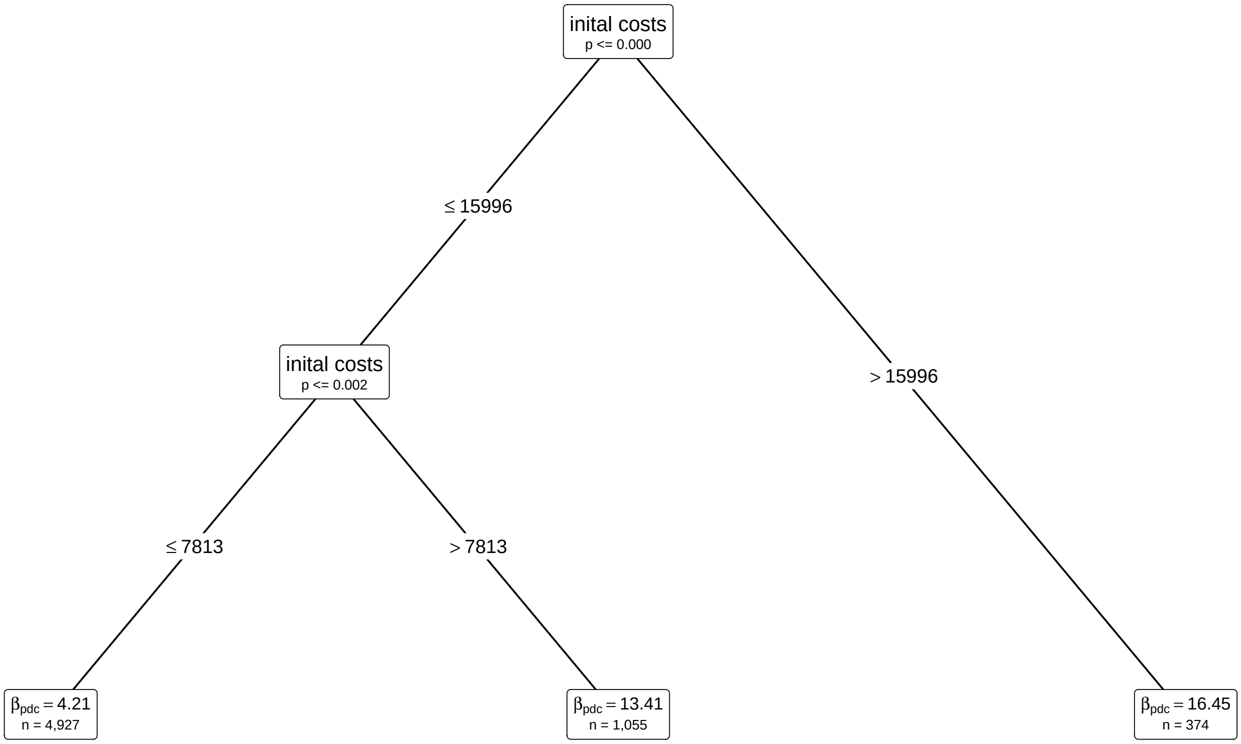

Figure 1: Model-based Decision Tree of Type 1 Diabetes with Stratified Effect Estimates (ß) of PDC.

Supplementary Figure 2

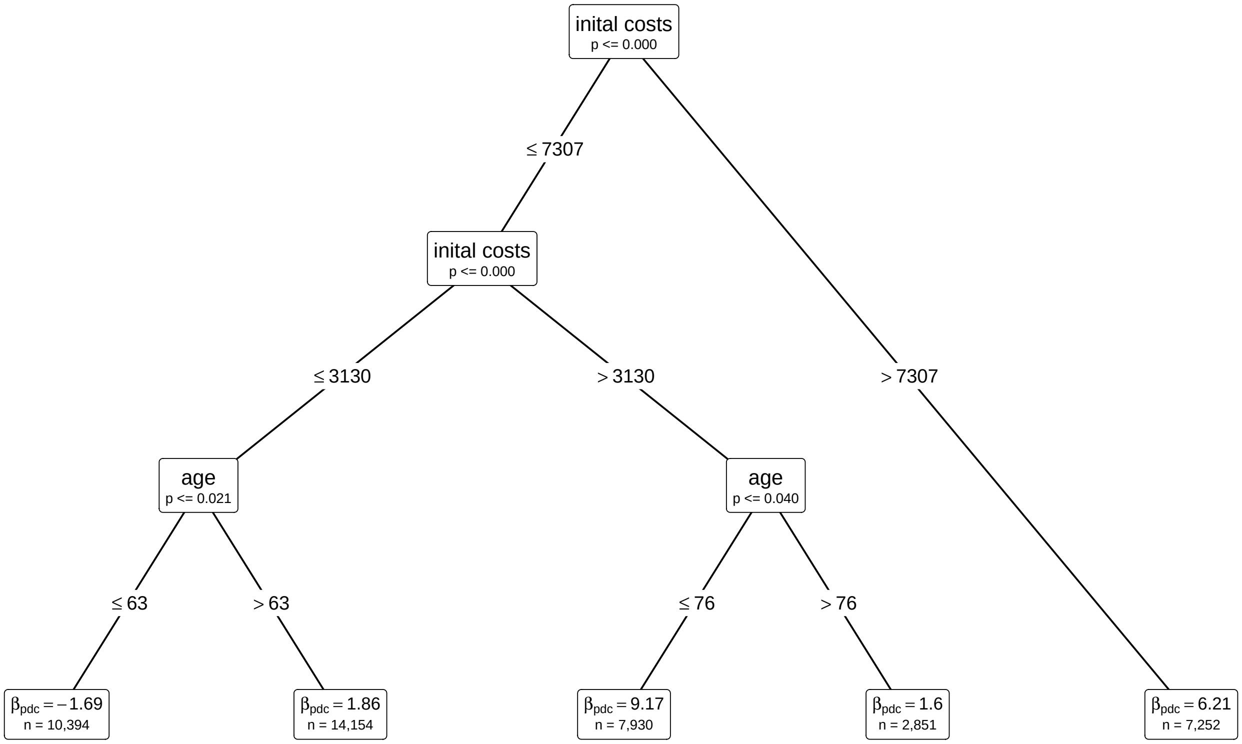

Figure 2: Model-based Decision Tree of Type 2 Diabetes with Stratified Effect Estimates (ß) of PDC.

Supplementary Figure 3

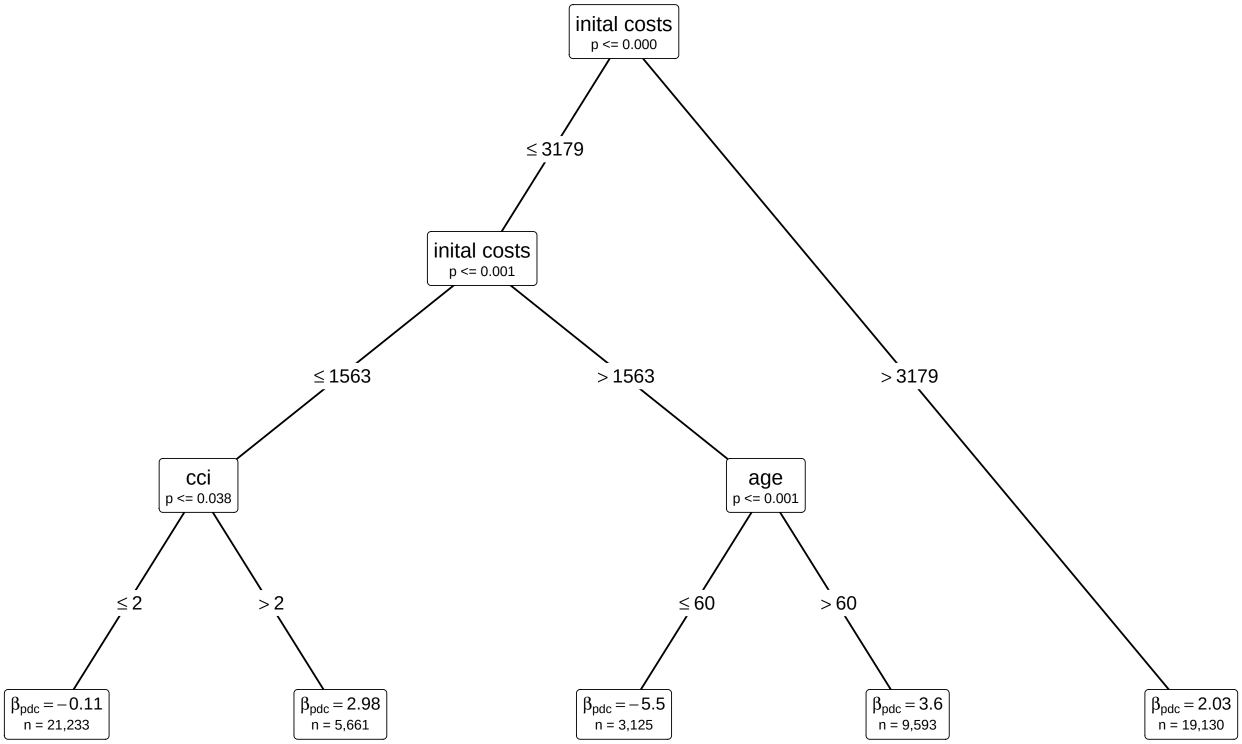

Figure 3: Model-based Decision Tree of Hyperlipidemia with Stratified Effect Estimates (ß) of PDC.

Supplementary Figure 4

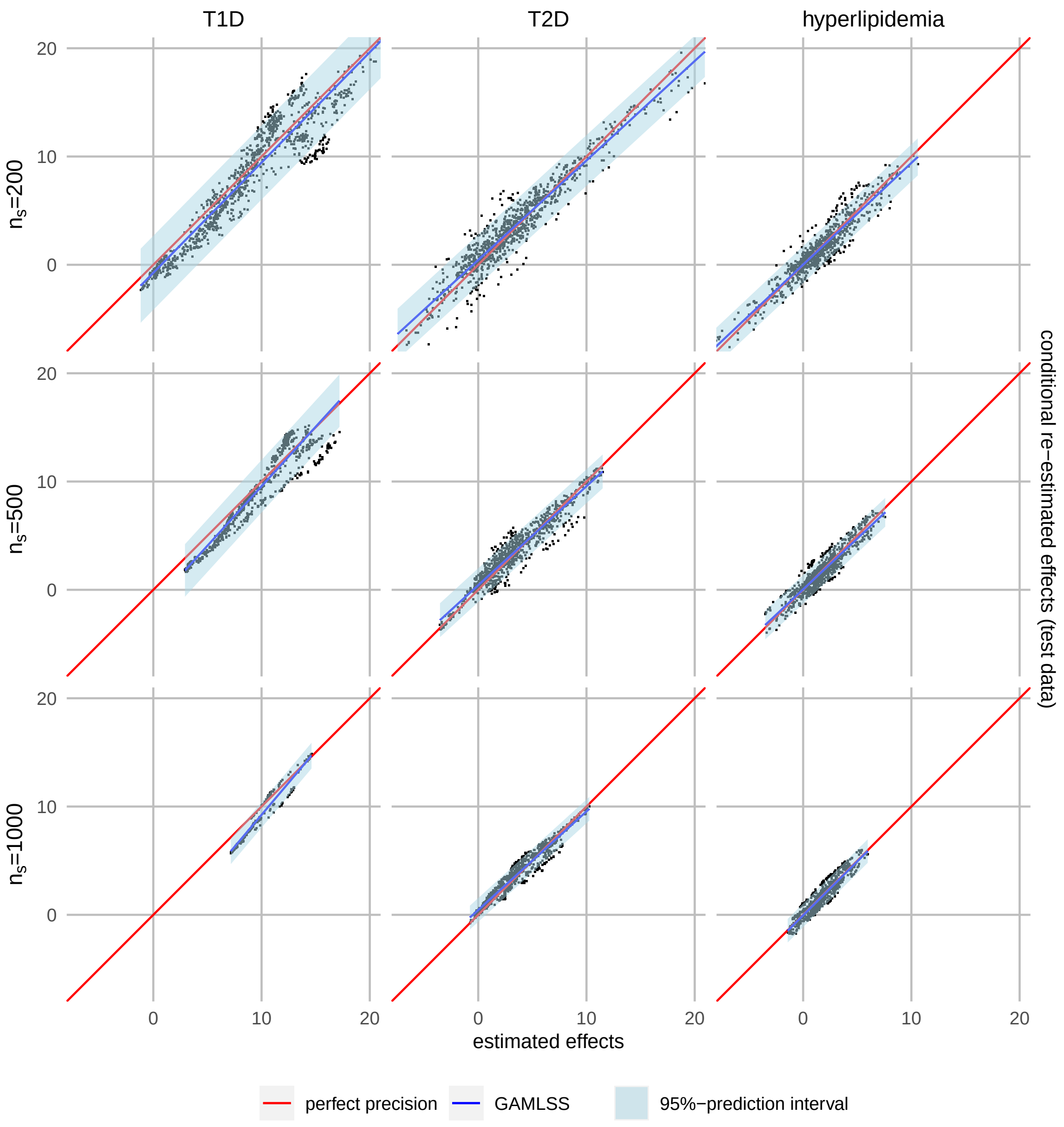

Figure 4: Calibration Plots with Linear Fit (Blue Line) and 95% Prediction Interval (Light Blue Area).

Supplementary Figure 5

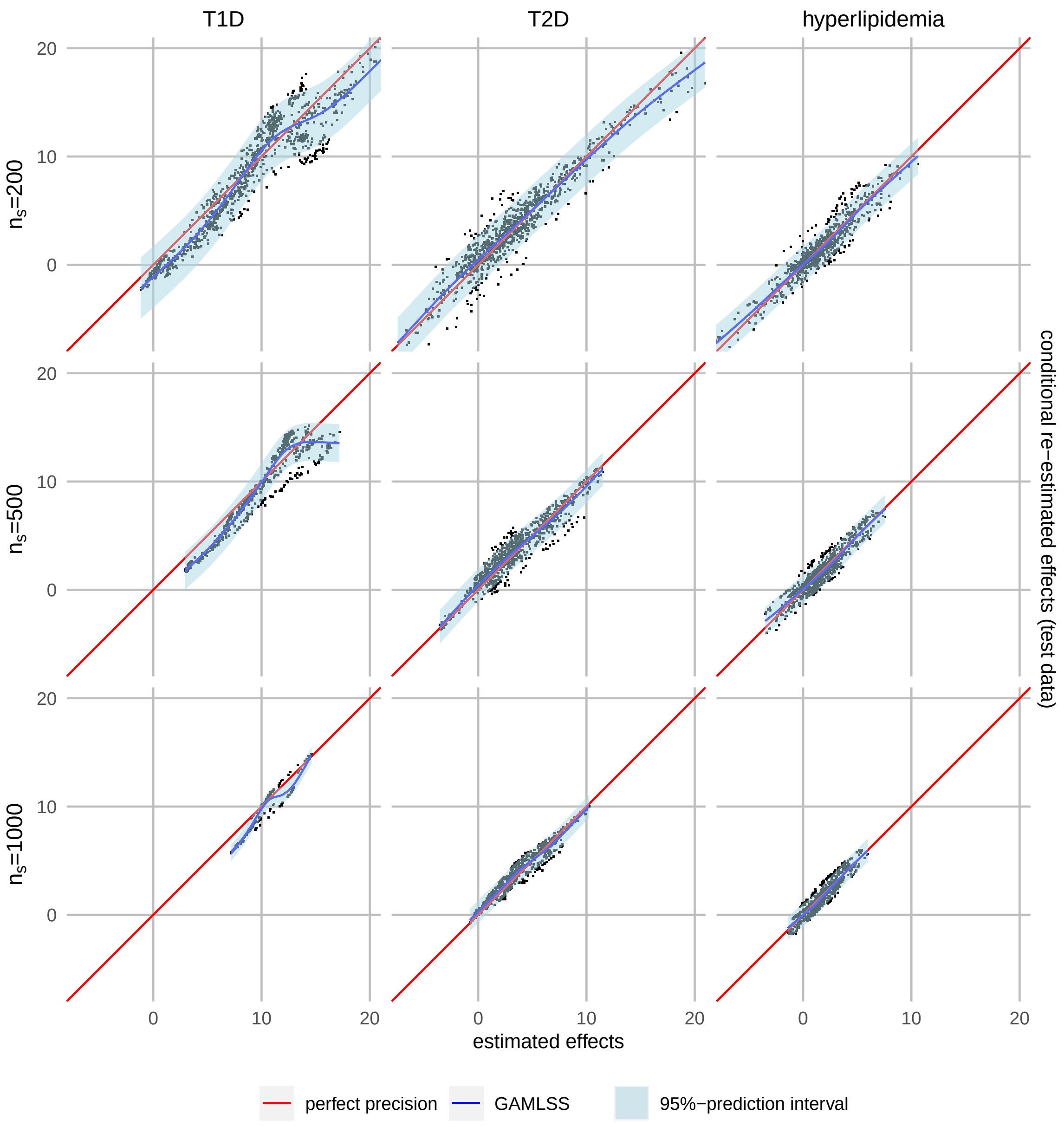

Figure 5: Calibration Plots with Non-Linear Fit (Blue Line) and 95% Prediction Interval (Light Blue Area).

Supplementary Figure 6

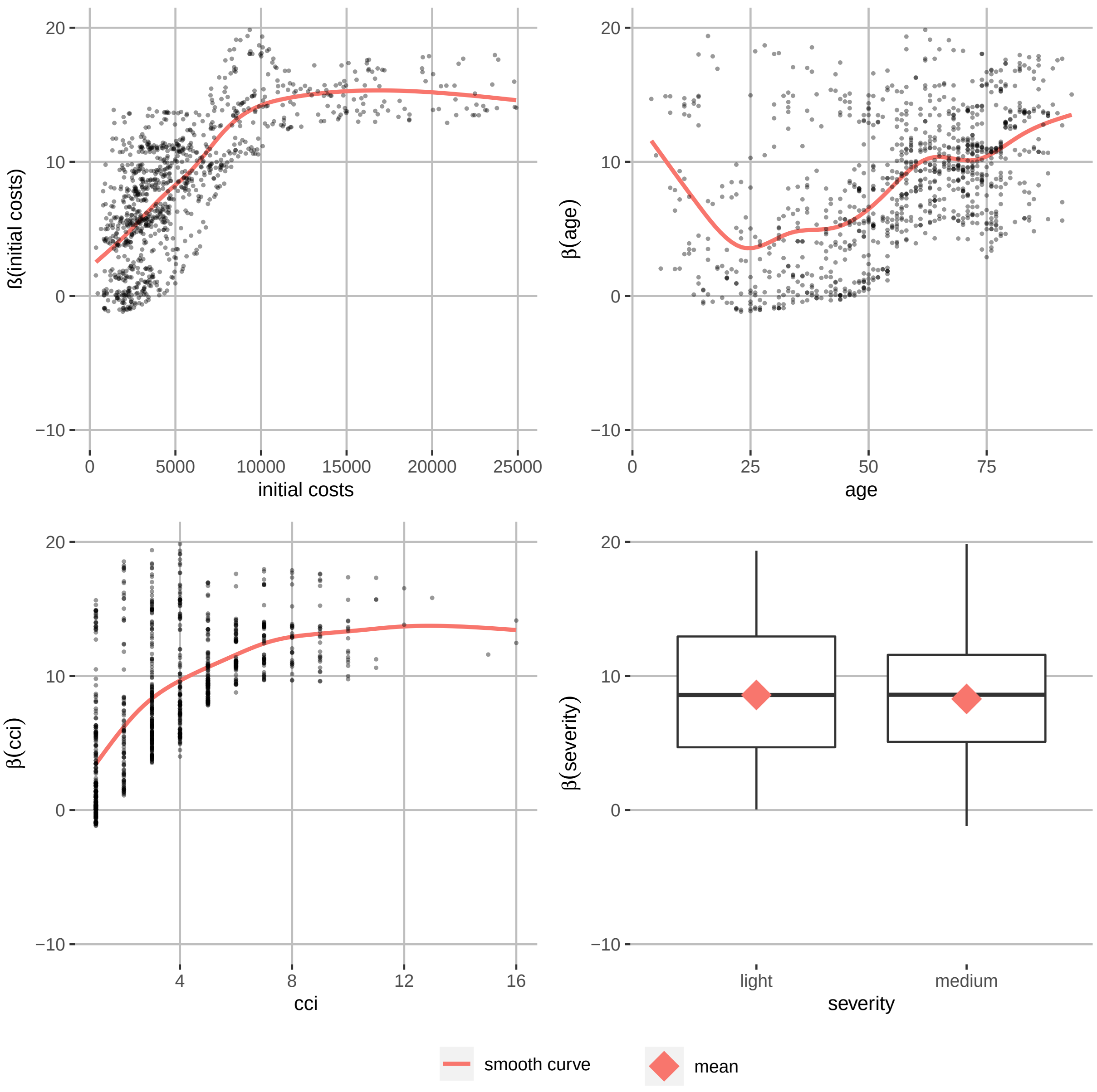

Figure 6: Partial Dependence Plots of T1D Patients with High Level of Similarity.

Supplementary Figure 7

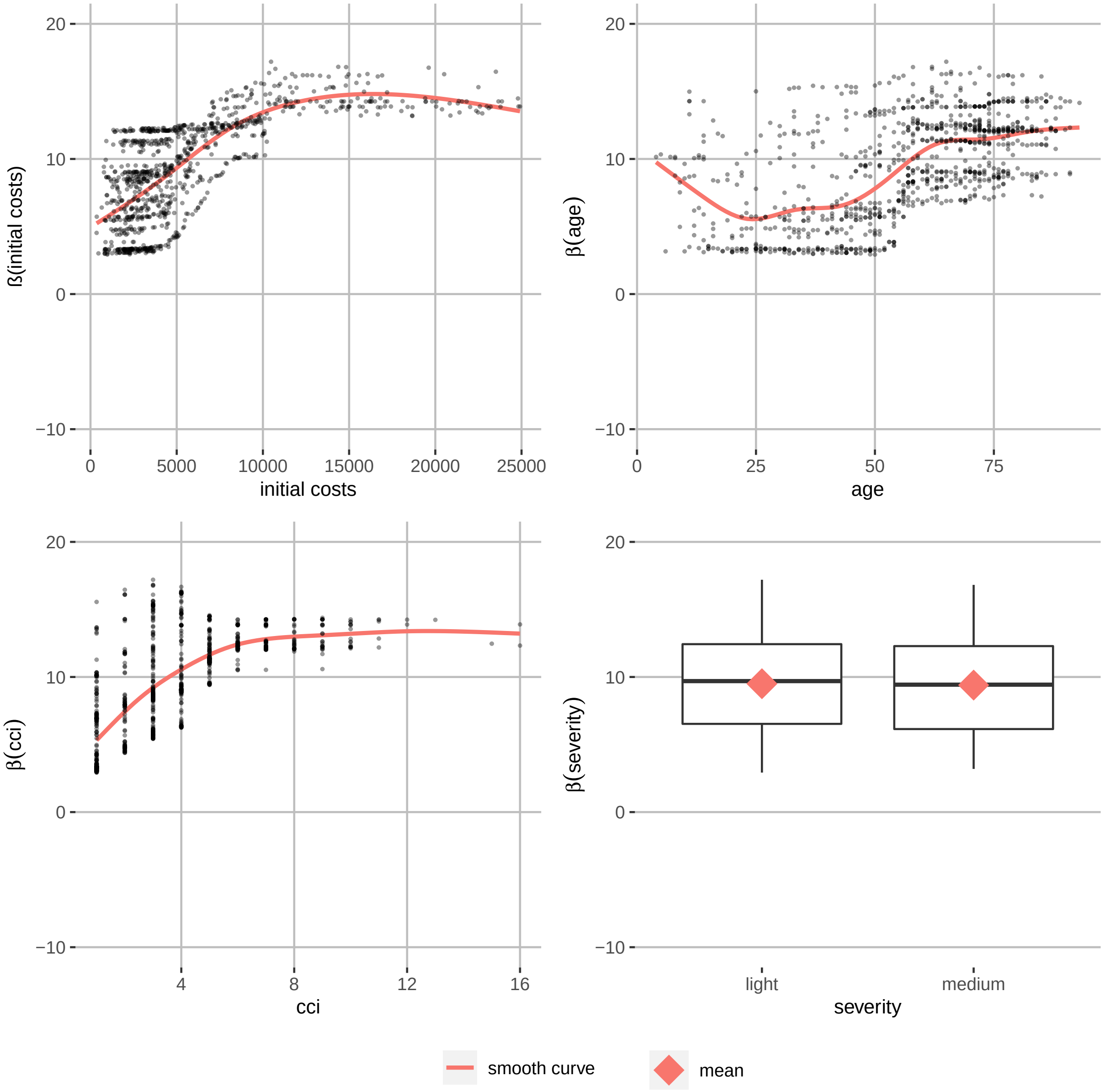

Figure 7: Partial Dependence Plots of T1D Patients with Medium Level of Similarity.

Supplementary Figure 8

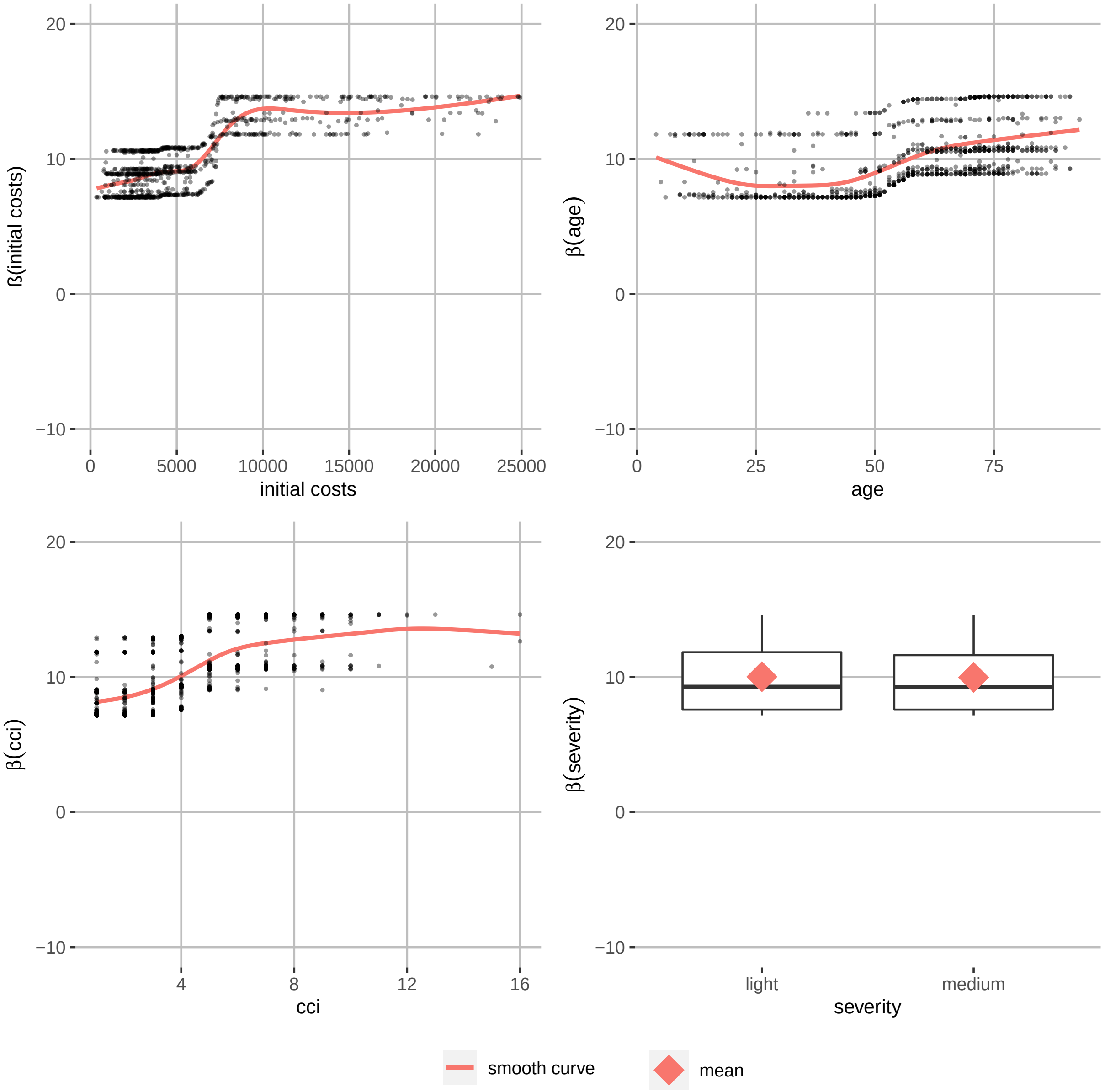

Figure 8: Partial Dependence Plots of T1D Patients with Low Level of Similarity.

Supplementary Figure 9

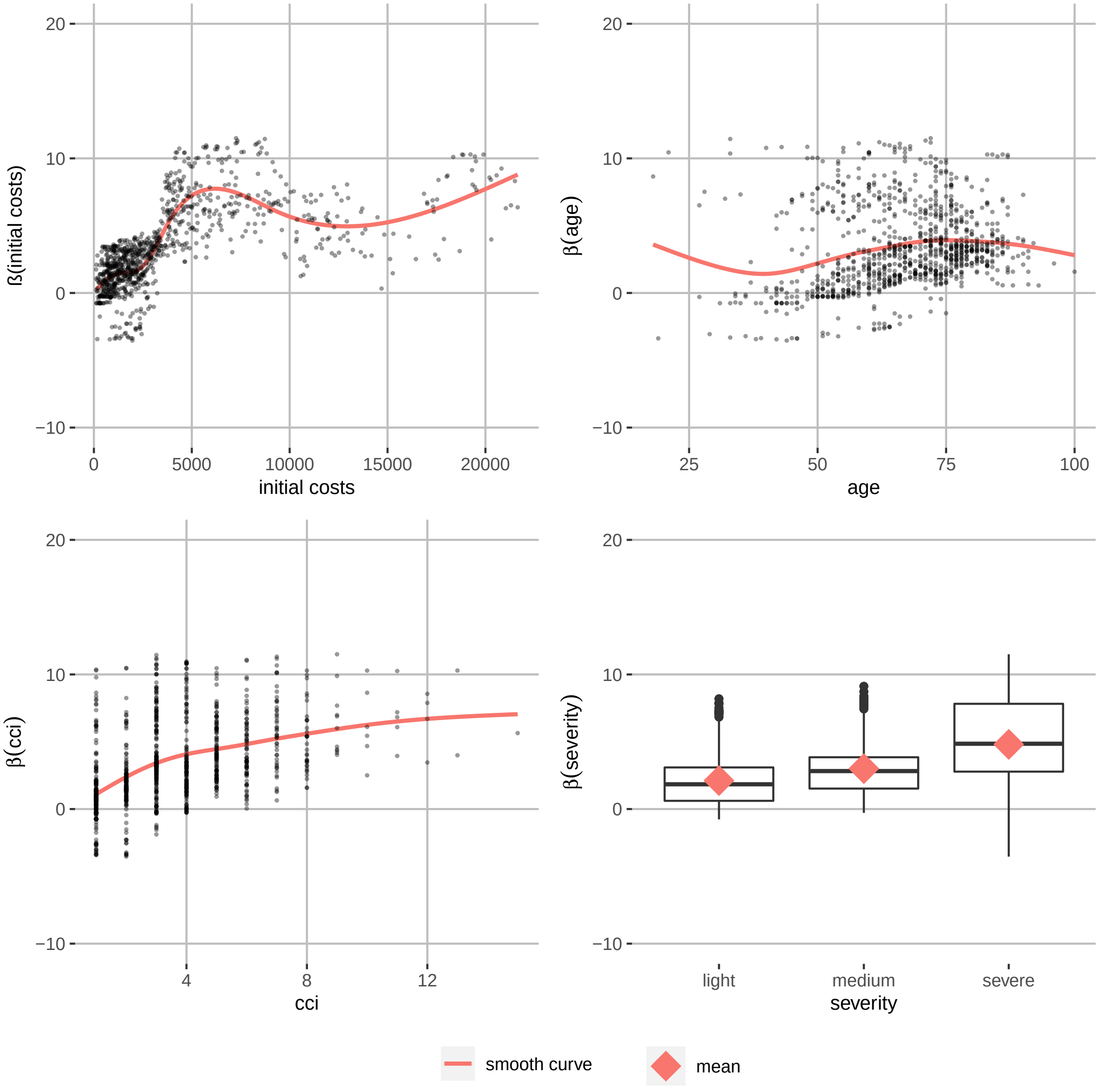

Figure 9: Partial Dependence Plots of T2D Patients with Medium Level of Similarity.

Supplementary Figure 10

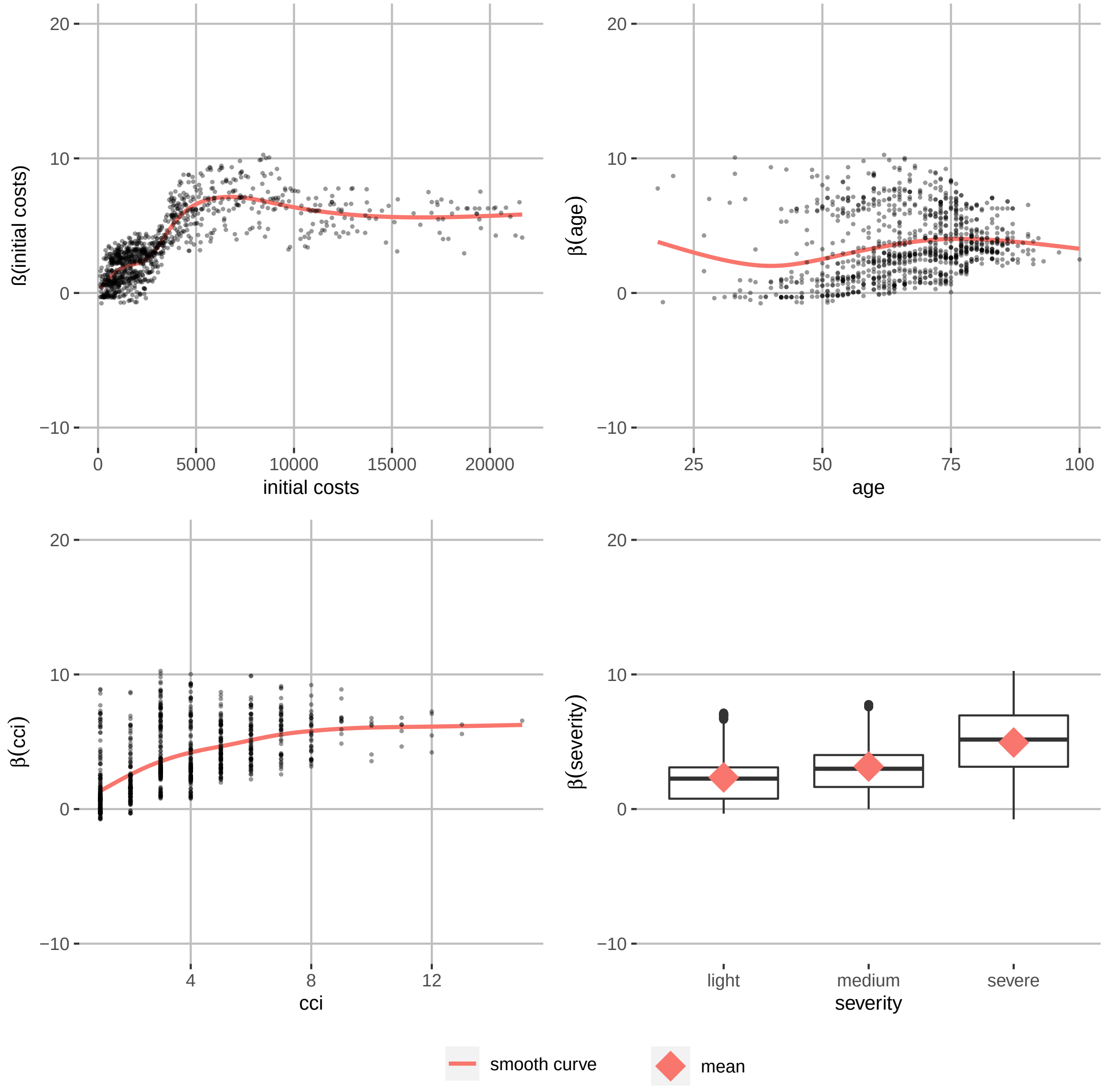

Figure 10: Partial Dependence Plots of T1D Patients with Low Level of Similarity.

Supplementary Figure 11

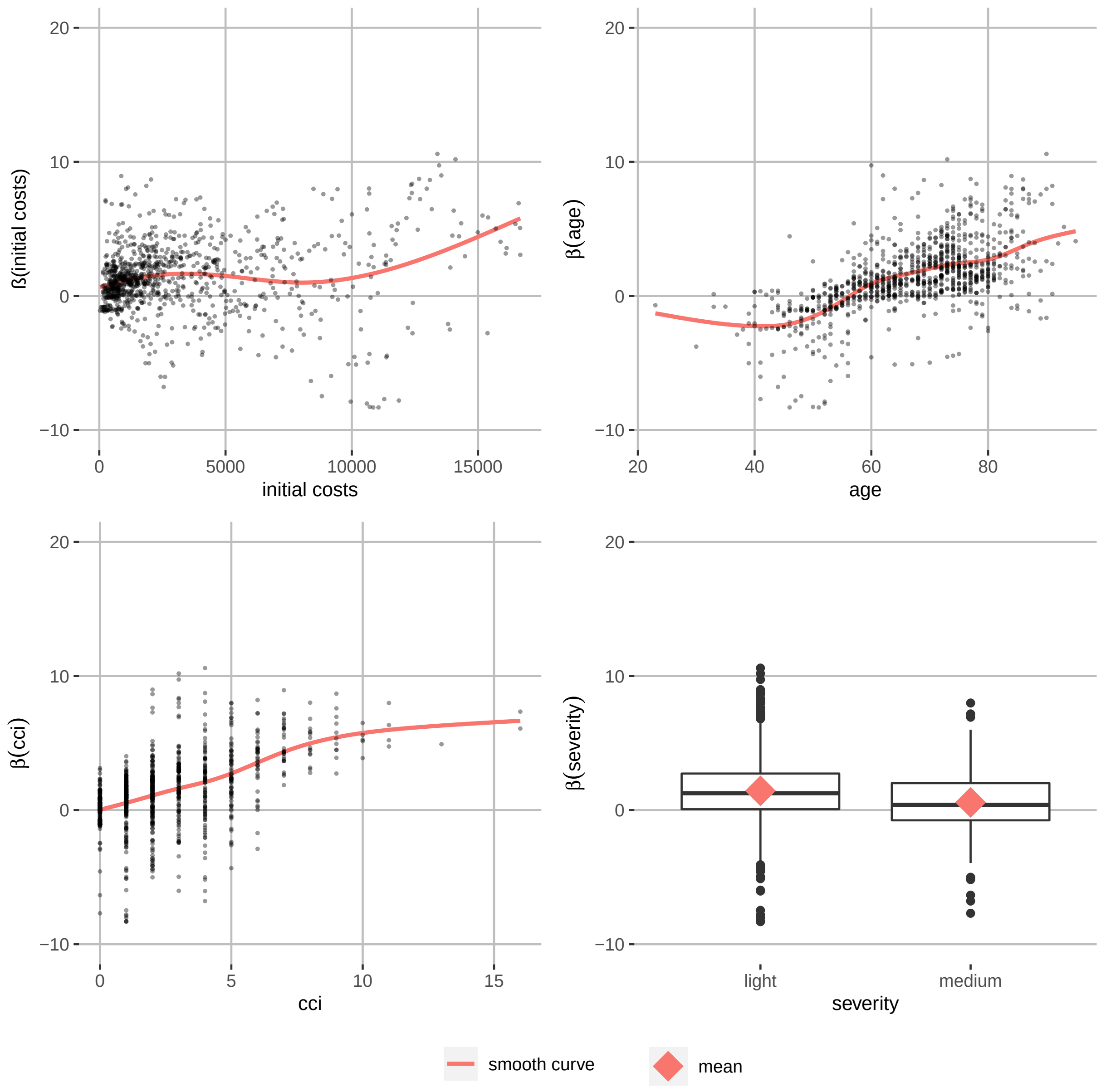

Figure 11: Partial Dependence Plots of Hyperlipidemia Patients with High Level of Similarity.

Supplementary Figure 12

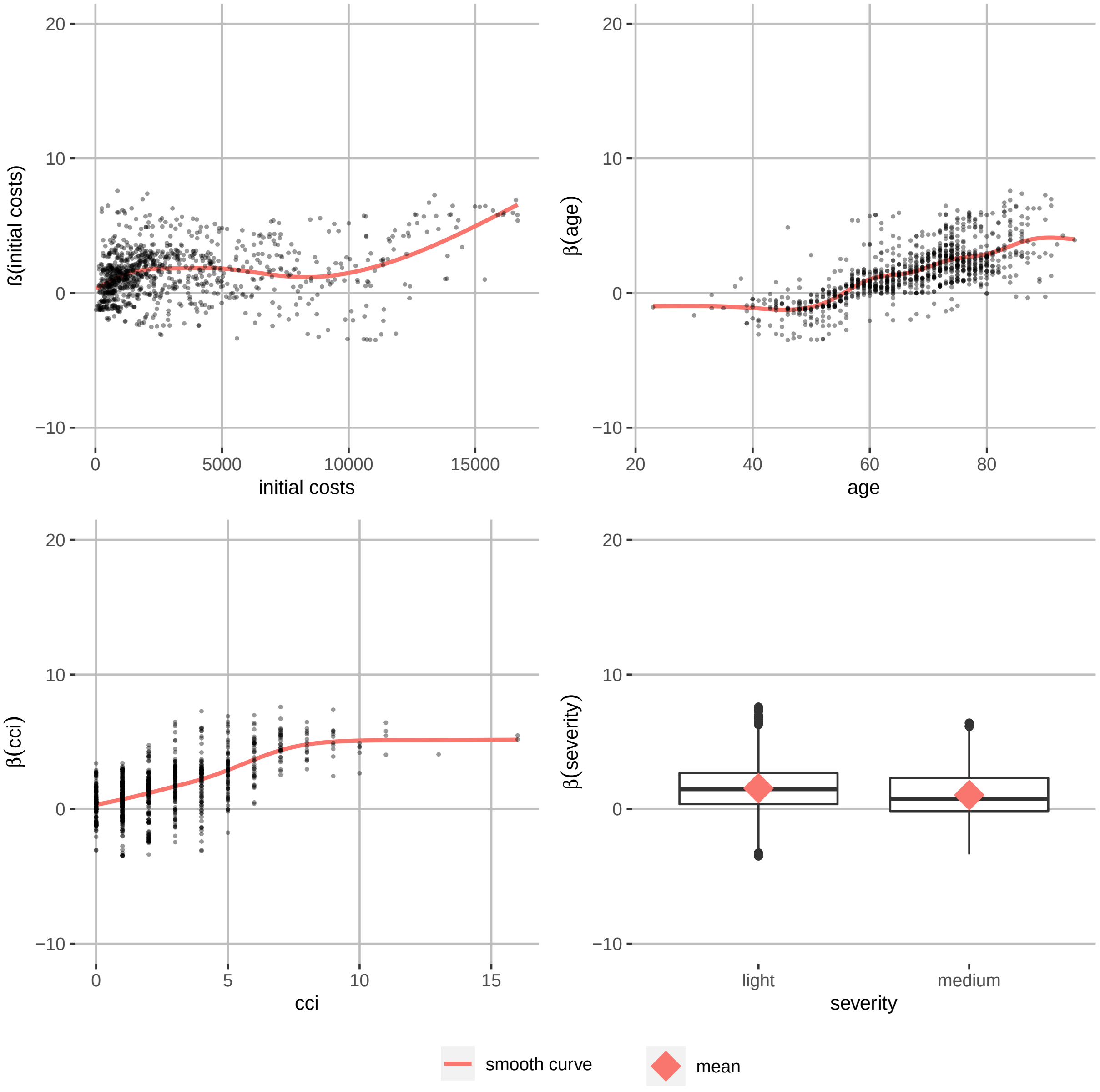

Figure 12: Partial Dependence Plots of Hyperlipidemia Patients with Medium Level of Similarity.

Supplementary Figure 13

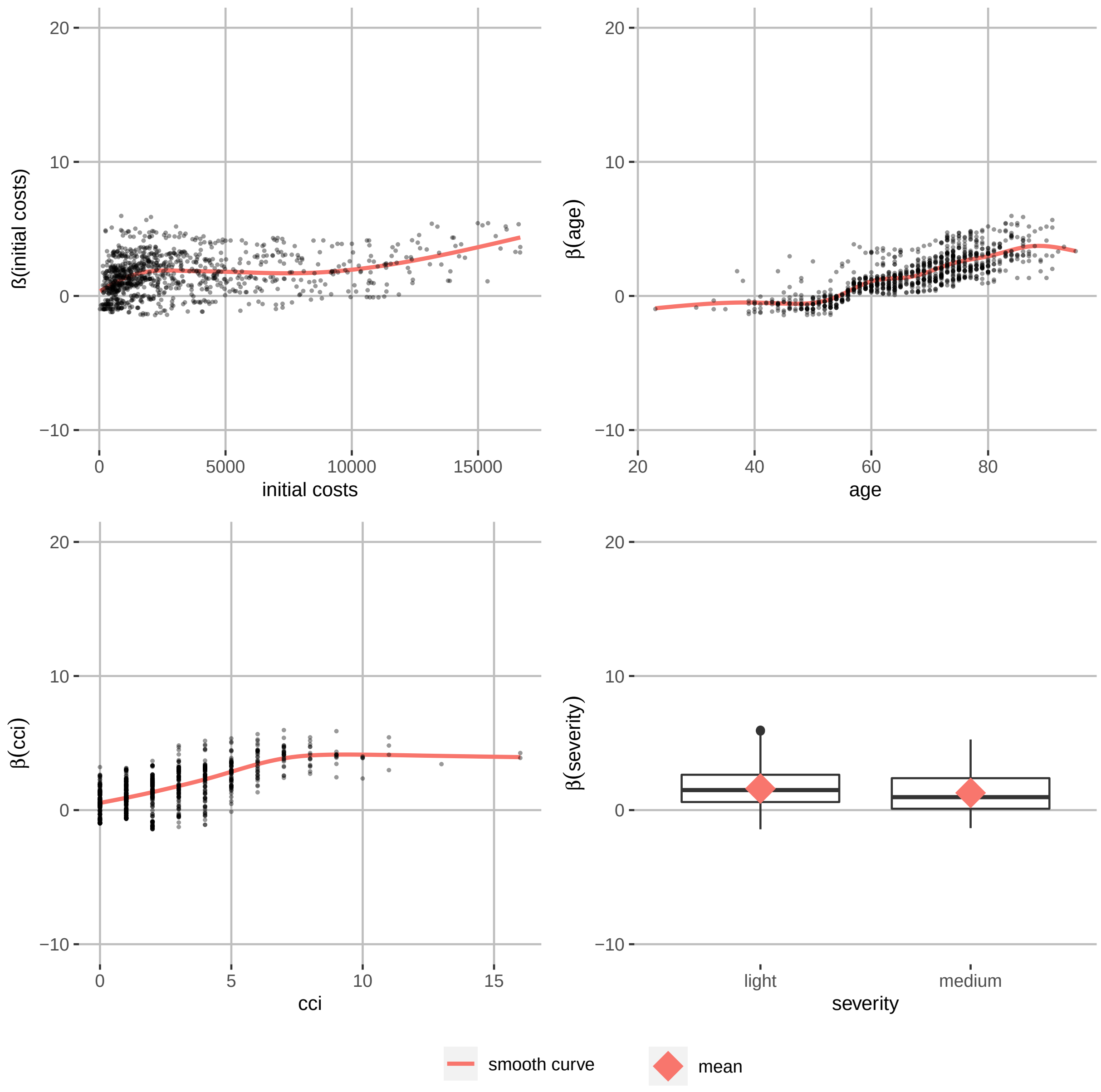

Figure 13: Partial Dependence Plots of Hyperlipidemia Patients with Low Level of Similarity.

References

Bundesärztekammer, Kassenärztliche Bundesvereinigung, and Arbeitsgemeinschaft der Wissenschaftlichen Medizinischen Fachgesellschaften. 2021. "Nationale Versorgungsleitlinie Typ-2-Diabetes: Teilpublikation Der Langfassung, 2. Auflage, Version 1." Edited by Bundesärztekammer, Kassenärztliche Bundesvereinigung, and Arbeitsgemeinschaft der Wissenschaftlichen Medizinischen Fachgesellschaften. https://doi.org/10.6101/AZQ/000475.

Deutsche Diabetes Gesellschaft. 2018. "S3-Leitlinie Therapie Des Typ-1-Diabetes: 2. Auflage." Edited by Deutsche Diabetes Gesellschaft.

Mach, François, Colin Baigent, Alberico L. Catapano, Konstantinos C. Koskinas, Manuela Casula, Lina Badimon, M. John Chapman, et al. 2020. "2019 Esc/Eas Guidelines for the Management of Dyslipidaemias: Lipid Modification to Reduce Cardiovascular Risk." *European Heart Journal* 41 (1): 111–88. https://doi.org/10.1093/eurheartj/ehz455.
